# Effects of karanjin on cell cycle arrest and apoptosis in human A549, HepG2 and HL-60 cancer cells

**DOI:** 10.1186/s40659-015-0031-x

**Published:** 2015-07-26

**Authors:** Jian-Ru Guo, Qian-Qian Chen, Christopher Wai-Kei Lam, Wei Zhang

**Affiliations:** State Key Laboratory of Quality Research in Chinese Medicines, Macau Institute for Applied Research in Medicine and Health, Macau University of Science and Technology, Taipa, Macau, China

**Keywords:** Karanjin, Cell cycle, Apoptosis, Cancer therapy

## Abstract

**Background:**

We have investigated the potential anticancer effects of karanjin, a principal furanoflavonol constituent of the Chinese medicine *Fordia cauliflora*, using cytotoxic assay, cell cycle arrest, and induction of apoptosis in three human cancer cell lines (A549, HepG2 and HL-60 cells).

**Results:**

MTT cytotoxic assay showed that karanjin could inhibit the proliferation and viability of all three cancer cells. The induction of cell cycle arrest was observed via a PI (propidium iodide)/RNase Staining Buffer detection kit and analyzed by flow cytometry: karanjin could dose-dependently induce cell cycle arrest at G2/M phase in the three cell lines. Cell apoptosis was assessed by Annexin V-FITC/PI staining: all three cancer cells treated with karanjin exhibited significantly increased apoptotic rates, especially in the percentage of late apoptosis cells.

**Conclusion:**

Karanjin can induce cancer cell death through cell cycle arrest and enhance apoptosis. This compound may be effective clinically for cancer pharmacotherapy.

## Background

*Fordia cauliflora* Hemsl, belongs to the family of Leguminosae and is, known in Chinese as “Shuiluosan”. It has been used in China as a traditional folk medicine for bronchitis, rheumatism, bruise, dementia of children, and valetudinarianism [1, 2]. Previous studies have shown that this plant could improve short and long-term memories of mice [3], with additional anti-hypertension [4], anti-inflammatory [5], and antioxidative effects [6].

**Figure 1:**
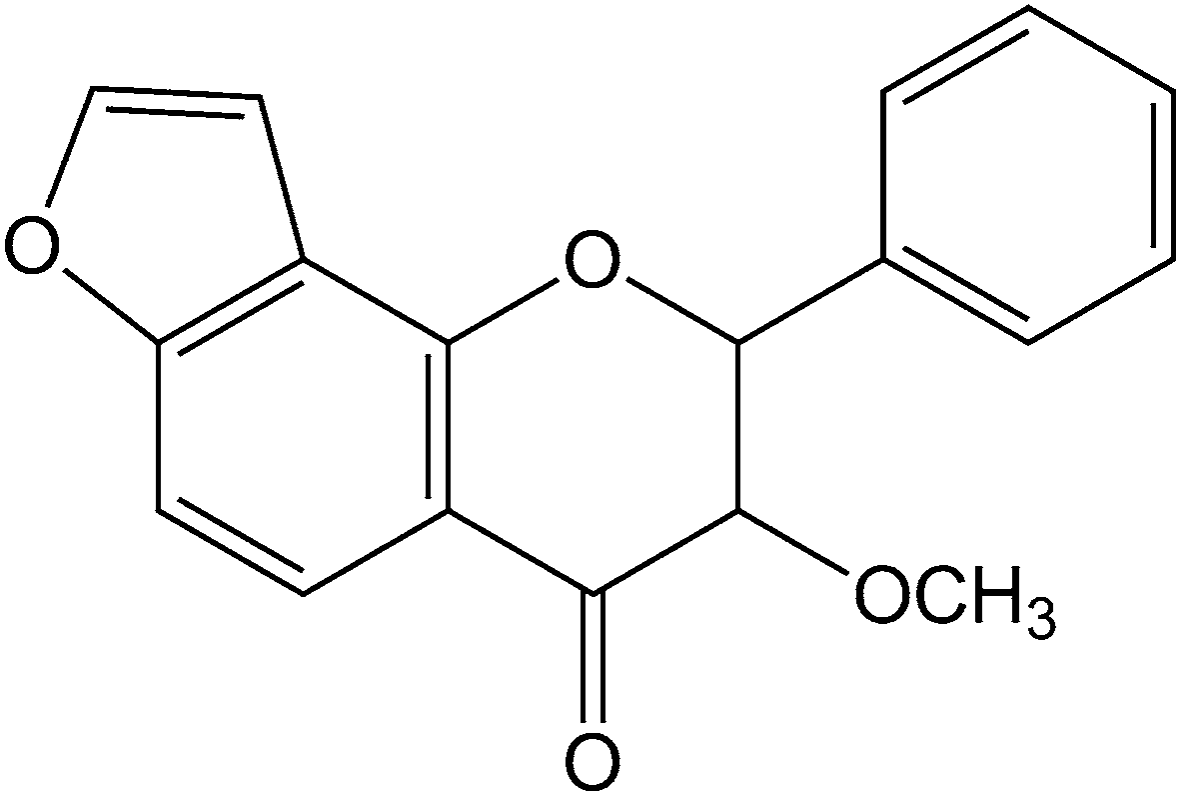
Chemical structure of karanjin.

Karanjin (structure depicted in Figure 1) is a major active furanoflavonol constituent of *Fordia cauliflora* Hemsl. It is associated with the above pharmacological activities, and has been reported to exert anti-hyperglycemic action [7] and ability of inducing GLUT4 translocation in skeletal muscle cells by increasing AMP-activated protein kinase activity [8]. In addition, Michaelis et al. found that karanjin could interfere with drug efflux mediated by ATP-binding cassette (ABC) transporters ABCB1, ABCC1, and ABCG2, and enhanced their ATPase activity. These mechanisms may be relevant to the anti-cancer effect of karanjin, especially in combination with other anti-cancer drugs that interfere with ABC transporters [9]. Flavonoids are widely distributed and used in traditional Chinese medicine for treating various diseases by virtue of their anticancer, antioxidant, antibacterial and anti-inflammatory activities. For example, Maurya et al. reported that bisfuranoflavonoids, furanorotenoids and dihydrofurano compounds have shown efficacy against human cancer cells [10]. Therefore it is logical to speculate that karanjin may also possess anti-cancer activity. However, to date this hypothesis has not been supported by any experimental evidence. In this study, karanjin isolated from *Fordia cauliflora* Hemsl. was investigated for its anti-tumor effects using cell cycle arrest and induction of apoptosis in three cancer cell lines: human lung adenocarcinoma cell line (A549), human hepatocellular carcinoma cell line (HepG2), and human acute promyelocytic leukemia cell line (HL-60 based mainly on our experience in working on them as well as the high prevalence and mortality of these three cancers in humans.

## Results and discussion

### Effect of Karanjin on growth of tumor cells

**Figure 2:**
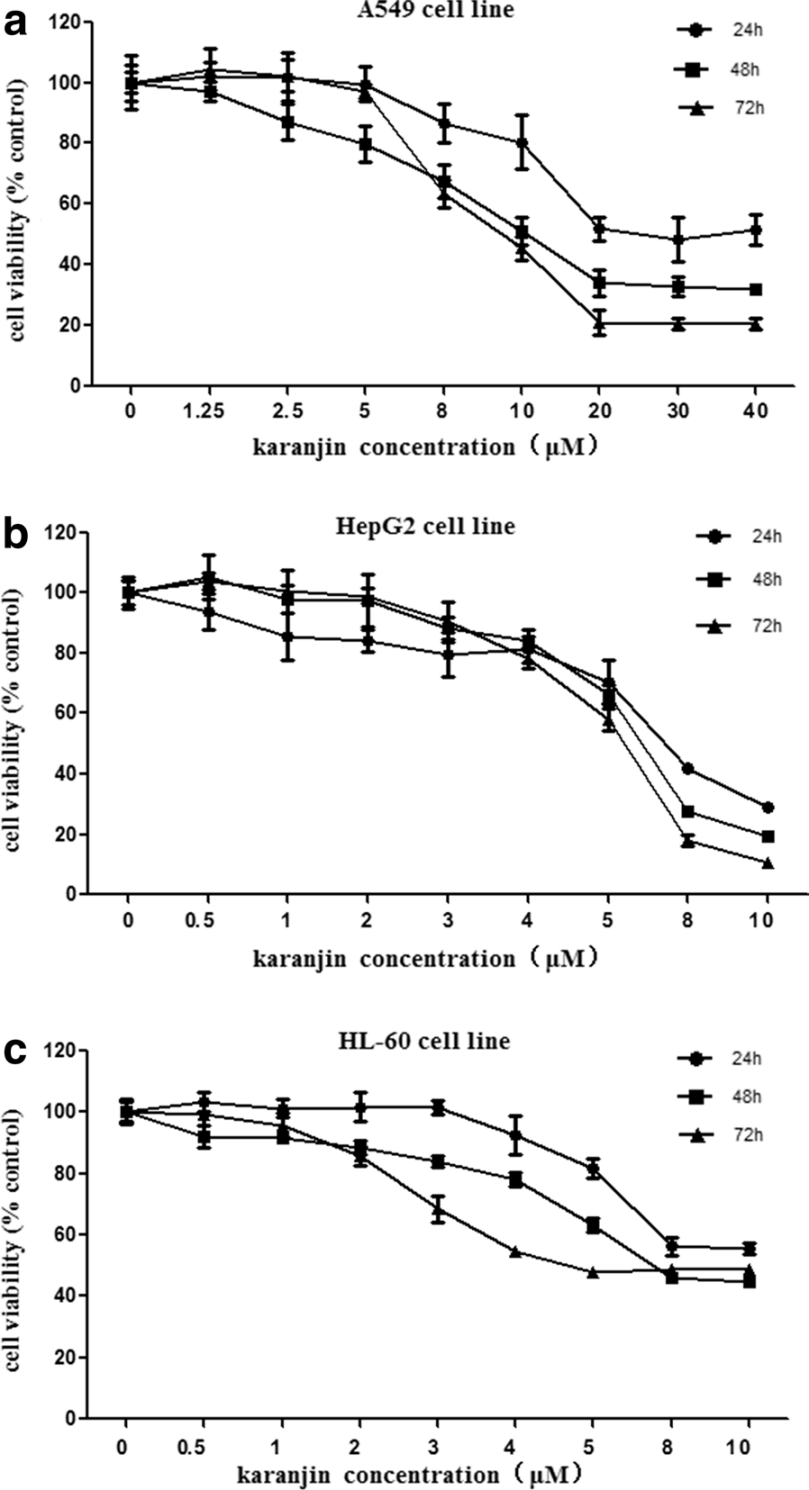
The cytotoxic effects of human cancer cell lines. MTT assay was performed after treatment with karanjin for 24, 48 and 72 h. **a** A549 cells, **b** HepG2 cells, and **c** HL-60 cells. All data are presented as mean ± SD values (n = 3).

Prompted by our interest in the antitumor activity of flavonoid compounds, we have investigated the cytotoxic effect and apoptotic property of karanjin in various human cancer cell lines A549, HepG2, and HL-60 representing respectively lung adenocarcinoma, hepatocarcinoma and promeylocytic leukemia that are prevalent with high mortality in humans. The inhibitory effect of karanjin on these cancer cell lines was determined by the cytotoxic MTT (3-[4,5-dimethylthiazol-2-yl]-2,5 diphenyltetrazolium bromide) assay. Cells were exposed to karanjin at various concentrations (Dr Zhang, please consider if you like to list the concentrations here in brackets like how you list the incubation times) over different incubation periods (24, 48 and 72 h). As shown in Figure 2, proliferation of karanjin-treated A549, HepG2 and HL-60 cells was significantly suppressed compared to untreated cells. The IC_50_ (half (50%) maximal inhibitory concentration) values of karanjin on these three cell lines over different treatment time periods (Table 1) showed that karanjin inhibition of A549, HepG2, HL-60 cells was time-dependent. IC_50_ values of karanjin-treated HepG2 cells were close to those of HL-60 cells over the same incubation times. However, IC_50_ values of A549 cells decreased steeply with increased drug exposure time (about 2.3- and 3.0-folds from 24 to 48 and 24 to 72 h), and they were higher than the corresponding IC_50_ values of HepG2 and HL-60 cells. These observation and comparison suggest that the HepG2 and HL-60 cell lines may be more sensitive to Karanjin with respect to viability and proliferation. Furthermore, we use gemcitabine, 5-fluorouracil (5-FU) and Cytosine Arabinoside (Ara-C) as positive-control anti-cancer agents for these three cell lines, respectively. These cytotoxic drugs have shown selective efficacy on the three types of cancer cells. IC_50_ values of A549 cells for treatment with gemcitabine over 72 h was 0.04 ± 0.01 µM, that of 5-FU on HepG2 cell line 49.9 ± 5.1 µM (72 h), and for Ara-C on HL-60 cells 2.6 ± 0.8 µM. We found that the cytostatic effect of karanjin was higher than 5-FU, but lower than gemctitabine and Ara-C. Even so, these results also could suggest that karanjin exhibited a strong inhibitory effect on these human cancer cells.

**Table 1:** IC_50_ values of karanjin in three cancer cell lines over different treatment times

Human cancer cell lines	IC_50_ value(µM)		
	24 h	48 h	72 h
A549	35.3 ± 5.3	15.1 ± 4.5	11.9 ± 3.1
HepG2	7.2 ± 0.7	6.2 ± 1.5	5.5 ± 1.3
HL-60	10.0 ± 1.4	8.1 ± 0.8	6.8 ± 0.9

### Karanjin induced cell cycle arrest at G2/M phase in cancer cell lines

**Figure 3:**
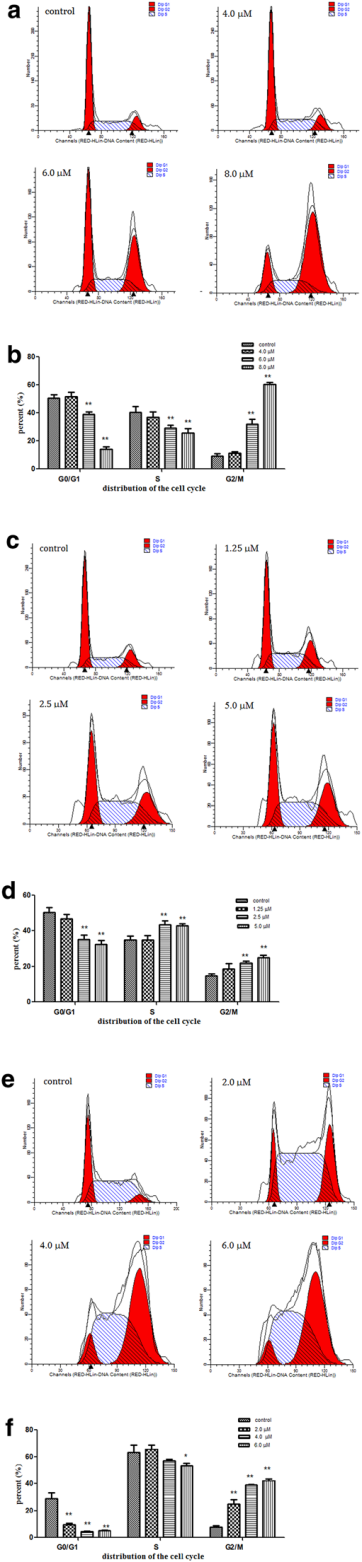
Karanjin induced cell cycle arrest at G2/M phase in cancer cell lines. **a** Effect of karanjin in A549 cells as analyzed using flow cytometry. **b** The percentages of A549 cells at different phases. **c** Effect of karanjin in HepG2 cells. **d** The percentages of HepG2 cells at different phases. **e** Effect of karanjin in HL-60 cells. **f** The percentages of HL-60 cells at different phases. All results are expressed as mean ± SD values from three independent experiments. *P* value of less than 0.05 (*P < 0.05, **P < 0.01, compared with the control group)are considered significant.

The cell cycle is a cyclic process of cell division. Accordingly, induction of cell cycle arrest may be a strategy for cancer therapy. Based on results of the above MTT assays, karanjin exhibited a potent inhibitory effect on the proliferation of all three human cancer cells that were studied. In order to investigate the effect of karanjin on the cell cycle phase distribution in these three cancer cell lines, PI (propidium iodide)/RNase Staining Buffer detection kit was used in the flow cytometric analysis. As shown in Figure 3a, b and Table 2, the percentage of A549 cells in G2/M phase increased dose-dependently upon 72 h treatment with karanjin, while S-phase cells showed moderate yet statistically significant decreases, and A549 cells in G0/G1 phase decreased. Figure 3c, d and Table 2 show that treatment of HepG2 cells with karanjin at 2.5 and 5.0 µM for 72 h resulted in decreased percentage of cells at G0/G1 phase, while cells of S and G2/M phases increased,. As shown in Figure 3e, f and Table 2, incubation with karanjin for 72 h could induce cell cycle arrest at G2/M phase in HL-60 cells dose-dependently from 2.0 to 4.0 and 6.0 µM, respectively. Our above results are similar to those of the other types of flavonoid compounds [11–13]. Karanjin could decrease the percentage of cells at G0/G1 and S phases and increase significantly the percentage of cells in the G2/M phase in A549 cells. It could inhibit cell growth by inducing the accumulation of S and G2/M phase cells in HepG2 cells. Exposure of HL-60 cells to karanjin caused cell cycle arrest at the G2/M phase, and a significantly decreased distribution of cells in the G0/G1 phase. Although these results indicate that karanjin could inhibit the grown of all three cancer cells through cell cycle arrest, HL-60 and A549 cells exhibited similar patterns of induction of cell cycle arrest. In summary, the cell cycle was markedly blocked at G2/M phase in A549 cells, moderately perturbed in HL-60 cells, and least arrested in HepG2 cells.

**Table 2:** Cell cycle phase distribution of A549, HepG2 and HL-60 cells with various doses of karanjin for 72 h

Cell line	Group	Cell cycle distribution (%)		
		G0/G1	S	G2/M
A549	Control	50.3 ± 2.0	40.5 ± 3.3	9.2 ± 1.5
	4.0 µM	51.6 ± 2.5	36.9 ± 3.0	11.2 ± 0.9
	6.0 µM	39.0 ± 1.4**	29.2 ± 1.7**	31.8 ± 2.8**
	8.0 µM	14.0 ± 1.5**	25.6 ± 2.6**	60.4 ± 1.2**
HepG2	Control	49.9 ± 2.1	34.3 ± 2.1	15.8 ± 0.1
	1.25 µM	46.8 ± 1.8	34.7 ± 2.0	18.5 ± 2.6
	2.5 µM	35.0 ± 2.2**	43.3 ± 1.8**	21.7 ± 1.0**
	5.0 µM	32.3 ± 1.8**	42.8 ± 1**	25 ± 1.0**
HL-60	Control	28.8 ± 3.6	63.1 ± 4.5	8.0 ± 0.8
	2.0 µM	9.6 ± 0.8**	65.5 ± 2.6	24.9 ± 2.7**
	4.0 µM	4.2 ± 0.5**	57.0 ± 0.9	38.8 ± 0.5**
	6.0 µM	4.9 ± 0.4**	53.0 ± 1.7*	42.1 ± 1.4**

Results are expressed as mean ± SD values from three independent experiments.

*P* value of less than 0.05 (* *P* < 0.05, ** *P* < 0.01, compared with the control group) are considered statistically significant.

### Karanjin induced apoptosis in cancer cells

**Figure 4:**
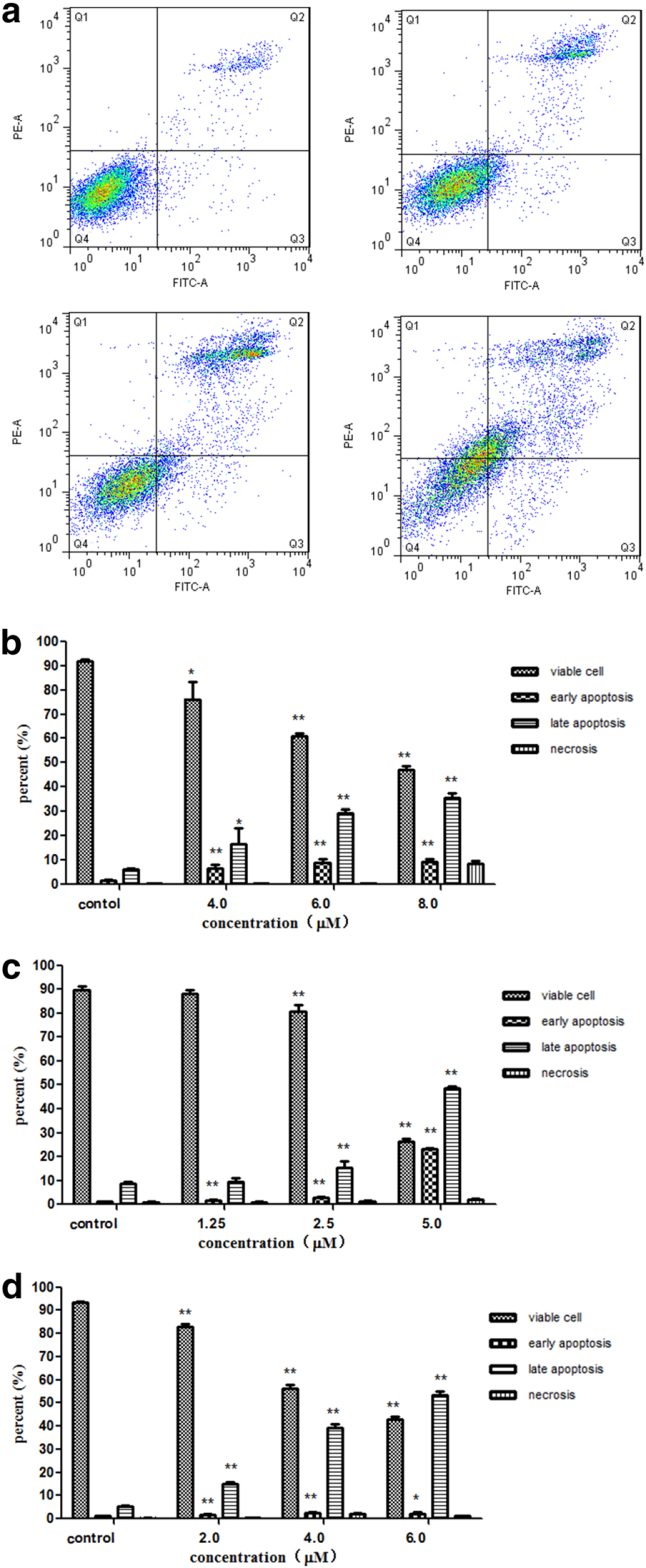
Karanjin induced apoptosis in cancer cell lines. **a** Annexin V-FITC/PI staining of A549 cells exposed to karanjin as analyzed by flow cytometry. **b** Data analyses of A549 cells. **c** Data analyses of HepG2 cells. **d** Date analyses of HL-60 cells. *Q1* necrosis, *Q2* late apoptosis, *Q3* early apoptosis, *Q4* viable cell. All results are expressed as mean ± SD values from three independent experiments. *P* value of less than 0.05 (*P < 0.05, **P < 0.01, compared with the control group)are considered significant.

Cell apoptosis is a normal physiological process of orderly controlled cell death for maintaining stable internal environment of the whole organism. There is evidence that apoptosis is related to cell cycle arrest [14]. Compounds that can induce cell cycle arrest and apoptotic cell death are generally considered to be potential anticancer drugs [15, 16]. The Annexin V-FITC/PI apoptosis detection assay was used to confirm if karanjin inhibited cell proliferation through apoptosis. As shown in Figure 4a, b and Table 3, treatment of A549 cancer cells with different doses of karanjin for 72 h resulted in significant increases in the ratios of early and late apoptosis cells, while the percentage of viable cells reduced. Figure 4c and Table 3 show that treatment of HepG2 cells with 5.0 µM karanjin for 72 h resulted in decrease of viable cells to 26.33 ± 0.90%, increase in early apoptosis cells to 23.23 ± 0.29%, and increase in late apoptosis to 48.43 ± 0.90% compared to untreated percentages (all P < 0.01). Figure 4d and Table 3 also show HL-60 cells displayed similarly high percentage of apoptosis cells upon treatment with karanjin for 72 h. The distribution of A549 and HL-60 apoptotic cells upon karanjin treatment belonged predominantly to those of late apoptosis. However, the accumulation of HepG2 apoptotic cells was not only in late apoptosis, but also in early apoptosis. In addition, we found that decreases in viable cells and increases in apoptotic cells were dependent on karanjin concentration. The percentages of late apoptosis in HepG2 and HL-60 cells are higher than that of A549 cells upon treatment with karanjin at high dose. Such different effects may be due to the differences between IC_50_ values. These experimental data provided evidence for karanjin induced apoptosis in A549, HepG2 and HL-60 cells, implying a strong correlation between inhibition of cell-proliferation and apoptosis. Many reports [17–19] have suggested the pathways of flavonoids for inducing apoptosis. The pathways for karanjin-induced apoptosis need to be further elucidated. Our study has shown that treatment of A549, HepG2 and HL-60 cells with karanjin caused inhibition of cell proliferation by increased cell apoptosis, suggesting that karanjin may be a potential drug for anti-cancer treatment.

**Table 3:** Effects of incubation with karanjin for 72 h on cell apoptosis in A549, HepG2 and HL-60 cells

Cell line	Group	Apoptosis distribution (%) in cells			
		Viable cell	Early apoptosis	Late apoptosis	Necrosis
A549	control	91.97 ± 0.34	1.59 ± 0.41	6.08 ± 0.36	0.35 ± 0.11
	4.0 µM	76.13 ± 5.99*	6.75 ± 1.04**	16.80 ± 5.08*	0.33 ± 0.02
	6.0 µM	61.03 ± 0.99**	9.03 ± 1.27**	29.47 ± 1.08**	0.44 ± 0.12
	8.0 µM	47.03 ± 1.43**	9.15 ± 0.92**	35.33 ± 1.72**	8.50 ± 0.92
HepG2	control	89.53 ± 1.37	1.16 ± 0.08	8.54 ± 0.83	0.79 ± 0.52
	1.25 µM	88.10 ± 1.21	1.68 ± 0.15**	9.29 ± 1.56	0.93 ± 0.29
	2.5 µM	80.83 ± 2.03**	2.70 ± 0.26**	15.40 ± 2.32**	1.07 ± 0.35
	5.0 µM	26.33 ± 0.90**	23.23 ± 0.29**	48.43 ± 0.90**	2.02 ± 0.21
HL-60	control	93.37 ± 0.33	1.10 ± 0.06	5.31 ± 0.22	0.23 ± 0.04
	2.0 µM	82.77 ± 0.99**	1.72 ± 0.21**	14.97 ± 0.70**	0.58 ± 0.07
	4.0 µM	56.33 ± 1.11**	2.38 ± 0.36**	39.40 ± 1.13**	1.93 ± 0.37
	6.0 µM	43.00 ± 1.02**	2.24 ± 0.59*	53.53 ± 1.13**	1.25 ± 0.11

Results are expressed as mean ± SD values from three independent experiments.

P-value of less than 0.05 (* P < 0.05, ** P < 0.01, compared with the control group) are considered statistically significant.

## Conclusion

In this study, the effects of flavonoid karanjin as a potential anticancer agent on the proliferation and apoptosis of human cancer cell lines including A549, HepG2 and HL-60 cells were investigated. The viability of these three cell lines was found to decrease both time and concentration dependently upon treatment with karanjin. In addition, all cell lines were shown to undergo accumulation of G2/M-phase late apoptosis cells dose-dependently upon treatment with karanjin for 72 h. These findings suggest that karanjin has significant anti-cancer activity on A549, HepG2 and HL-60 cells by inducing cell cycle arrest and apoptosis.

## Methods

### Chemicals and reagents

The Karanjin used in this study was a gift from Professor Buming Liu at the Guangxi Key Laboratory of Traditional Chinese Medicine Quality Standards, Guangxi, China. Karanjin was isolated from plant *Fordia cauliflora* by repeated open-column chromatography and preparative reverse-phase high pressure liquid chromatography. Its structure was determined by UV, MS, 1H- and 13C-NMR, and by comparison with literature values [20, 21]. Ultra-pure water was obtained from the Milli-Q Gradient Water System (Millipore Corporation, MA, USA). Human lung adenocarcinoma cell line A549, human hepatocellular carcinoma cell line HepG2, and human acute promyelocytic leukemia cell line HL-60 were purchased from American Type Culture Collection (ATCC), Rockville, MD, USA. For culturing cells, phosphate buffer saline (PBS), Dulbecco’s Modified Eagele Medium (DMEM), RPMI medium 1640, 0.25% Trypsin–EDTA solution, penicillin–streptomycin solution and fetal bovine serum (FBS) were purchased from GIBCO Invitrogen Corp, Carlsbad, CA, USA. 3-[(4,5)-dimethylthiazol-2-yl]-2,5-diphenyl tetrazolium bromide (MTT) was purchased from Sigma Aldrich Chemical Co., St Louis, MO, USA. Cell Cycle Analysis Kit and Annexin V-FITC/PI Apoptosis Detection Kit were purchased from Signalway Antibody (SAB) Co., Ltd. College Park, MD, USA.

### Cell culture

The human HepG2 cells was cultured in DMEM medium with 10% FBS, 100 units/mL penicillin, 100 μg/mL streptomycin in a 37°C humidified incubator with 5% CO_2_ atmosphere, A549 cells and HL-60 cells were cultured in RPMI Medium 1640 supplemented with 10% FBS, 100 UI/mL penicillin and 100 μg/mL streptomycin in humidified air at 37°C with 5% CO_2_, respectively. The exponentially growing cells were collected and re-suspended in fresh media and then exposed to different concentrations of karanjin.

### MTT assay

The inhibition of cell growth by karanjin was investigated via the MTT assay [22]. A549 cells, HepG2 cells and HL-60 cells were seeded in 96 wells plate (LabServ, Thermo Fisher Scientific, Bei Jing, China) at 3 × 10^3^ cells/well. After incubation, they were treated with the karanjin at different concentrations for 24, 48 and 72 h. MTT solution (final concentration of 0.5 mg/mL in medium) was added to each well and incubated further for 4 h. The medium was removed and 100 μL of DMSO was added to each well to dissolve the purple crystals of formazan. Absorbance was measured at 570 nm with a microplate UV/VIS spectrophotometer (Infinite M200 PRO, Tecan Auatria GmbH 5082, Grödig, Auatria); reference wavelength was 650 nm. The cell number was determined using a hemocytometer. IC_50_ values of karanjin were calculated by GraphPad Prism software. Cell viability (%) = OD_treated_/OD_control (untreated)_ × 100.

### Cell cycle analysis

The cell cycle analysis was determined as previously described [23]. Cells were seeded at about 2 × 10^4^ cells/well in six wells culture plates (LabServ, Thermo Fisher Scientific, Bei Jing, China) and duplicated wells were treated with karanjin at different concentrations for 72 h. Cells were then harvested and fixed in 70% (v/v) cold ethanol at 4°C overnight. After washing with ice-cold PBS, the fixed-cell pellets were collected by centrifugation and re-suspended in PI/RNase Staining Buffer (Cell Cycle Detection Kit, Signalway Antibody (SAB) Co. Ltd.) for staining of DNA and finally analysed on a flow cytometer (FACSAriaTM III; Beckon Dickinson and Co., B.D Biosciences, San Jose, CA, USA).

### Cell apoptosis analysis

Apoptosis was quantified by the Annexin V-FITC assay [24]. Cells treated with different concentrations of karanjin for 72 h were harvested by trypsinization, washed twice with 4°C PBS, and re-suspended in binding buffer. Annexin V-FITC and Propidium iodide (PI) solution were then added to stain the cells before analysis by flow cytometry (Beckon Dickinson FACSAriaTM III flow cytometer).

### Statistical analysis

Data analyses were performed using the IBM SPSS Statistics (International Business Machines Corp. Armonk, New York, USA) and GraphPad Prism softwares (GraphPad Software, Inc., La Jolla, CA, USA). All results are expressed as mean ± SD values from three independent replicate experiments. *P* value of less than 0.05 (*P < 0.05, **P < 0.01, compared with the control group)are considered to be statistically significant by using one-way ANOVA followed by the Student’s *t* test.

## References

[1] Liang ZY, Yang XS, Wang Y, Hao XJ, Sun QY (2010). Two new chalcones from *Fordia cauliflora*. Chin Chem Lett..

[2] Fan L, Zhang Y, Huang R, Qin S, Yi T, Xu F (2013). Determination of five flavonoids in different parts of *Fordia cauliflora* by ultra performance liquid chromatography/triple-quadrupole mass spectrometry and chemical comparison with the root of *Millettia pulchra* var. *laxior*. Chem Cent J..

[3] Li ZQ (2002). Study on effecte of *Fordia cauliflora* on mouse acquired memory disorder. Acad J Guangdong Coll Pharm..

[4] Huang RB, Jiao Y, Jiang WZ, Duan XQ, Kong XL, Yang ZH (2003). Effect of Yulangsan extract on the cardiac hemodynamics and the coronary flow in rats [J]. Chin Hosp Pharm J..

[5] Tang ZQ, Chen BS, Zhou Z, Wu ZQ, Qiu CC, Chen SF (2003). Anti-inflammatory effect of various extracts of *Fordia cauliflora*. Chin J of Ethnomed Ethnopharm..

[6] Wu ZQ, Zhou Z, Wei QZ (2004). Protective effects of Abstracts of *Fordia Cauliflora* Hemsl on bromobenzene-induced oxidative liver damage in mice and antioxidative capabil ity in old mice. Chin Pharmacol Bull..

[7] Tamrakar AK, Yadav PP, Tiwari P, Maurya R, Srivastava AK (2008). Identification of pongamol and karanjin as lead compounds with antihyperglycemic activity from *Pongamia pinnata* fruits. Etnopharmacol..

[8] Jaiswal N, Yadav PP, Maurya R, Srivastava AK, Tamrakar AK (2011). Karanjin from *Pongamia pinnata* induces GLUT4 translocation in skeletal muscle cells in a phosphatidylinositol-3-kinase-independent manner. Eur J Pharmacol..

[9] Michaelis M, Rothweiler F, Nerreter T, Sharifi M, Ghafourian T, Cinatl J (2014). Karanjin interferes with ABCB1, ABCC1, and ABCG2. J Pharm Pharm Sci..

[10] Maurya R, Yadav PP (2005). Furanoflavonoids: an overview (review). Nat Prod Rep.

[11] Tanigawa S, Fujii M, Hou DX (2008). Stabilization of p53 is involved in quercetin-induced cell cycle arrest and apoptosis in HepG2 cells. Biosci Biotechnol Biochem..

[12] Kang TB, Liang NC (1997). Studies on the inhibitory effects of quercetin on the growth of HL-60 leukemia cells. Biochem Pharmacol..

[13] Kuo PC, Liu HF, Chao JI (2004). Survivin and p53 modulate quercetin-induced cell growth inhibition and apoptosis in human lung carcinoma cells. J Biol Chem..

[14] Green DR (1998). Apoptotic pathways: the roads to ruin. Cell..

[15] Elmore S (2007). Apoptosis: a review of programmed cell death. Toxicol Pathol..

[16] Vermeulen K, Van Bockstaele DR, Berneman ZN (2005). Apoptosis: mechanisms and relevance in cancer. Ann Hematol..

[17] Nguyen TT, Tran E, Nguyen TH, Do PT, Huynh TH, Huynh H (2004). The role of activated MEK-ERK pathway in quercetin-induced growth inhibition and apoptosis in A549 lung cancer cells. Carcinogenesis..

[18] Qiao Y, Xiang Q, Yuan L, Xu L, Liu Z, Liu X (2013). Herbacetin induces apoptosis in HepG2 cells: Involvements of ROS and PI3K/Akt pathway. Food Chem Toxicol..

[19] Lowe SW, Lin AW (2000). Apoptosis in cancer. Carcinogenesis..

[20] Vismaya, Eipeson WS, Manjunatha JR, Srinivas P, Sindhu Kanya TC (2010). Extraction and recovery of karanjin: a value addition to karanja (*Pongamia pinnata*) seed oil. Ind Crop Prod..

[21] Katekhaye SD, Kale MS, Laddha KS (2012). A simple and improved method for isolation of karanjin from *pongamia pinnata* Linn seed oil. Indian J Nat Prod Resour..

[22] Sobottka SB, Berger MR (1992). Assessment of antineoplastic agents by MTT assay: partial underestimation of antiproliferative properties. Cancer Chemother Pharmacol..

[23] Vindelov LL, Christensen IJ, Nissen NI (1983). Standardization of high-resolution flow cytometric DNA analysis by the simultaneous use of chicken and trout red blood cells as internal reference standards. Cytometry..

[24] Zhang G, Gurtu V, Kain SR, Yan G (1997). Early detection of apoptosis using a fluorescent conjugate of Annexin V. Biotechniques..

